# Study of the solid–gas–stress coupling model and its application

**DOI:** 10.1038/s41598-022-24273-8

**Published:** 2023-03-29

**Authors:** Xianzhi Shi, Dazhao Song

**Affiliations:** 1 Xichang University, Xichang, Sichuan China; 2 Guizhou Yuxiang Mining Group Investment Co. Ltd., Bijie, Guizhou China; 3 School of Civil and Resource Engineering, University of Science and Technology Beijing, Beijing, China

**Keywords:** Natural hazards, Solid Earth sciences

## Abstract

Coal mines may change from non-outburst mines into coal and gas outburst mines with increasing mining depth. Therefore, scientific and rapid prediction of the coal seam outburst risk and effective prevention and control measures could ensure coal mine safety and production. This study aimed to propose a solid–gas–stress coupling model and assessed its applicability in predicting the coal seam outburst risk. Based on a large amount of outburst case data and the research results of previous scholars, coal and coal seam gas constitute the material basis of outbursts, and gas pressure is the energy source of coal seam outbursts. A solid–gas–stress coupling model was proposed, and a solid–gas–stress coupling equation was established via regression. Among the three major outburst factors, the sensitivity to the gas content during outbursts was the lowest. The causes of coal seam outbursts with a low gas content and the effect of the structure on outbursts were explained. It was theoretically revealed that the coupling of the coal firmness coefficient, gas content and gas pressure determined whether coal seams could experience outbursts. This paper provided a basis for assessing coal seam outbursts and classifying outburst mine types and listed application examples of solid–gas–stress theory.

## Introduction

The coal seam is characterized by heterogeneity and low permeability, and the gas is enclosed in the coal seam pores^1^, with increasing coal mining depth, the coal seam gas content and gas pressure also show an increasing trend. An increasing number of coal mines have developed from non-coal and non-gas outburst (non-outburst) mines into coal and gas outburst mines. Different scholars have different views on the influencing factors of coal and gas outbursts. The influence of the gas content^2,3^, gas pressure (in situ stress)^4–6^, structural coal^7,8^ and the combination of more than two factors^9,10^ on outbursts has been increasingly recognized in tests, practice and theory. Scholars believe that the energy source of coal and gas outbursts is the gas pressure, and they have quantitatively studied the outburst energy from the aspect of the gas elastic potential^11–13^. In the above research results, there is insufficient understanding of the material basis of coal and gas outbursts, and it is not clear whether structural coal and coal seam gas constitute the material basis of outbursts^2,3,14^ or the source of the outburst energy^15,16^. Regarding the classification of the coal seam outburst risk (outburst risk), the Detailed Rules for the Prevention and Control of Coal and Gas Outbursts^17^ mainly adopts four coal seam outburst risk identification indicators (coal seam gas pressure P, coal firmness coefficient f, coal damage type, and initial speed of coal gas emission Δp). The indices of the coal seam gas pressure and gas content are typically used for outburst prediction. Wu et al.^18^ and Ma et al.^19^ used 6 indicators to divide the coal seam outburst risk, with many evaluation indicators and difficult collection conditions. Gui et al.^20^ used grey system theory to establish an outburst prediction model to predict the coal seam outburst risk.

Although the above scholars have studied the factors leading to coal seam outburst, and some scholars have conducted the coupling of binary factors^9^, so far there is no theory to couple the gas pressure as the power source of coal and gas outburst with three factors such as coal seam firmness and gas content, establish a multivariate coupling model, calculate the coal seam outburst intensity, and then classify the coal mine outburst types. The Detailed Rules for the Prevention and Control of Coal and Gas Outburst and the above scholars have predicted the outburst using different indicators, but they all need many indicators (for example, 6 indicators are required in the literature^19^), and some indicators are difficult to collect, even requiring special construction roadways to collect. Some prediction methods are complicated and difficult to master^19,20^. With the occurrence of outburst accidents in low gas mines and coal seams with low gas content, the above research results are difficult to reasonably explain. In order to further clarify the problems that have not been solved by the above scholars, the purpose of this study was to propose a solid–gas–stress coupling model and evaluate its applicability in predicting the coal seam outburst risk. To achieve this goal, the following procedures were performed: by collecting a large number of case data of coal and gas outbursts near rock roadways and crosscuts, near faults and before the implementation of regional outburst prevention measures, this paper studied the coupling effect of various coal seam outburst factors, established a coupling relationship between the structural coal firmness coefficient, coal seam gas content and gas pressure (stress) and outburst coal quantity and the critical conditions of outbursts, and expounded the causes of low-content gas coal seam outbursts. An index for the division of outburst coal seams and outburst mine types was proposed.

## Methods

### Study design

**Figure 1:**
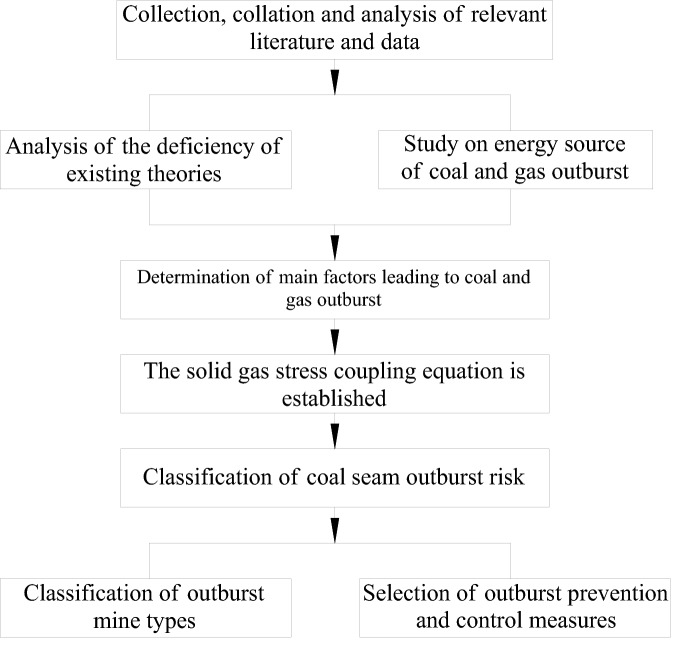
Flowchart of this study.

The flowchart of this study is shown in Fig. 1. Specifically, by collecting a large amount of literature and data pertaining to coal and gas outburst cases near rock roadways, crosscuts, and faults before the implementation of regional outburst prevention measures on a large scale, the shortcomings of existing outburst prevention theories were analysed, and the primary energy source of coal seam outbursts was identified as the gas elastic potential. By using the regression analysis method, the three main factors that lead to coal seam outbursts were determined, and a solid–gas–stress coupling equation was established. With the use of this equation and statistical data, the outburst risk of coal seams and the types of outburst mines were categorized according to the amount of outburst coal. Moreover, targeting different types of outbursts, different prevention measures were proposed.

### Data collection and sorting

Coal and gas outburst, referred to as outburst, is a phenomenon involving the sudden emission of coal and gas in a structural coal seam containing gas under the action of the gas pressure (in situ stress)^21,22^. Structural coal and gas constitute the material basis of outbursts, and the gas pressure (in situ stress) is the energy source of outbursts^6,15,16^. Practice, experimental evidence and statistical data of outburst-prone coal seam mining^2–7,9–11,16^ show that coal and gas outbursts are the result of the comprehensive action of the gas pressure (in situ stress), coal seam gas content and coal body strength. Among these factors, the coal seam damage type is the external manifestation of coal body strength reduction and is one of the inherent characteristics of coal seams. The coal seam damage degree reflects the coal body strength. The geological structure exhibits in situ stress concentration^14,22,23^, and the tectonic stress in coal seams is ultimately manifested as the coal seam gas pressure and the damage degree of coal seams (i.e., the coal seam firmness coefficient f). Therefore, the coupling of the three basic indicators, i.e., the coal seam firmness coefficient f, coal seam gas content W and coal seam gas pressure P, affects the coal outburst volume, i.e., the outburst intensity^16^.

To study the effect of the coal seam gas pressure, coal seam gas content and coal seam firmness coefficient on the outburst risk degree of outburst coal seams, data on a large number of rock roadway outburst cases, crosscut coal uncovering outburst cases, and fault zone outburst cases and outburst case data pertaining to coal mining-heading faces before the implementation of regional outburst prevention measures across China were collected (Guizhou^10^, Shanxi^21^, Anhui^24^, Hunan, Jiangxi, Sichuan^24,25^, Henan^26^, et al.), referring to the outburst data for the Donbas Gagarin coal mine in the former Soviet Union^25^. The coal outburst amount in each case was determined, and gas geological parameters (coal seam gas pressure, coal seam gas content and coal seam firmness coefficient) of the corresponding mines were collected. The statistical data are listed in Table 1.

**Table 1:** Statistics of the outburst coal quantity and gas geological parameters.

Serial number	Coal mine	Region (country)	Gas pressure P (MPa)	Gas content W (m^3^/t)	Coal seam firmness coefficient f	Outburst coal quantity Y (t)
1	Wulunshan Coal Mine	Guizhou	2.22	17.4	0.37	889
2			2.08	16.69	0.47	450
3	Xintian Coal Mine		2.5	25	0.2	2599
4	Xinhua Coal Mine		1.64	18.4	0.37	1010
5	Jinjia Coal Mine		1.8	12.1	0.22	1571
6	Xiangshui Coal Mine		1.23	12	0.31	2500
7	Anshun Coal Mine		1.7	8.9	0.24	571
8			0.92	9.2	0.91	85
9	Yutianbao Coal Mine	Sichuang	5.01	25	0.18	8765
10	Moxinpo Coal Mine		4.4	11.29	0.17	5270
11	Donglin Coal Mine		4.95	24	0.18	3109
12	Tonghua Coal Mine	Chongqing	2.4	23.32	0.32	3000
13	Yongsha Coal Mine	Jiangxi	3	35.3	0.18	3200
14	Shangzhuang Coal Mine		1.27	9.11	0.3	318
15	Luling Coal Mine	Anhui	4.1	21.75	0.17	10,500
16	Wangfenggang Coal Mine		1.8	21.7	0.29	2831
17	Lier Coal Mine		0.7	4.6	0.73	110
18	Kongli Coal Mine		0.66	5.2	0.73	170
19	Xieer Coal Mine		1.53	15.2	0.61	507
20			3.87	14.39	0.21	1014
21	Daping Coal Mine	Henan	2	11.7	0.12	1894
22	Yanmazhuang Coal Mine		2.44	20	0.43	1500
23	Longshan Coal Mine		1.19	16.6	0.98	315
24	Gedian Coal Mine		0.62	12.1	0.484	36
25	Hebi Liu Coal Mine		0.76	9.5	0.33	130
26	Hongling Coal Mine	Liaoning	6.2	22.5	0.26	5390
27	Aiheshan Coal Mine	Hunan	1.72	13	0.2	1021
28	Tongzishan Coal Mine		2.07	15	0.24	1267
29	Longjiashan Coal Mine		1.63	12	0.26	1420
30	Gagarin Coal Mine	Former Soviet Union	5	20	0.14	14,500

### Theoretical research

#### Solid–stress coupling elastic potential of coal seams

The elastic potential (potential energy) is the deformation of an object that can perform external work upon restoration to the original state, and energy is thus provided. The elastic potential is related to the degree of deformation of an object. The greater the deformation is, the higher the potential energy. Under the action of a high stress level, gas stored in the coal seam occurs in the form of the elastic potential energy in the coal seam gap. The energy stored in gas is directly related to the stress. The higher the gas pressure in the coal seam is, the higher the stored elastic potential energy of the coal seam^11,13,25^. There are two kinds of stored elastic potential energy, namely, coal deformation and gas in coal^25^.

**Figure 2:**
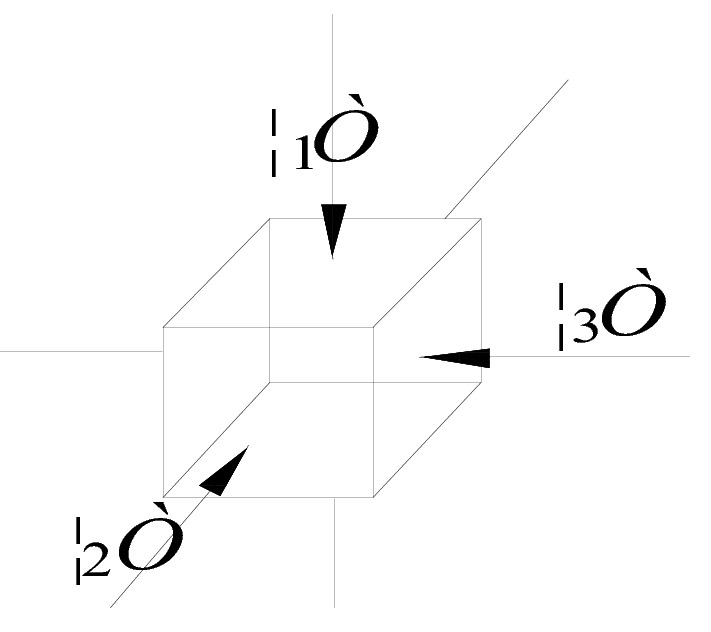
Schematic diagram of the principal stress distribution at a certain point in coal body.

The elastic potential of the coal seam is all the energy stored per unit volume of coal during elastic straining of the coal body. According to the von Mises yield criterion and distortion energy density theory, in the three-dimensional stress state, the strain energy of an elastomer and the work performed by the external force are equal in value, and the magnitude depends on the final value of the external force and deformation but is unrelated to the force adding process^27^. According to this theory, the elastic potential of coal is half of the product sum of the principal stress and strain. The principal stress distribution at a certain point in the coal body is shown in Fig. 2. Please refer to Eqs. (1–6) for the calculation process of the elastic potential of the coal body.

The elastic potential of coal can be calculated as follows:

1 $$ W_{t} = \frac{1}{2}(\sigma_{1} \varepsilon_{1} + \sigma_{2} \varepsilon_{2} + \sigma_{3} \varepsilon_{3} ) $$

Of which:

Equation (1) can be expressed as^25^:

2 $$ \varepsilon_{1} = \frac{1}{{\text{E}}}[\sigma_{1} - {\text{u}}(\sigma_{2} + \sigma_{3} )] $$

3 $$ {\text{W}}_{{\text{t}}} = \frac{1}{{2{\text{E}}}}[\sigma_{{^{1} }}^{2} + \sigma_{{^{2} }}^{2} + \sigma_{3}^{2} - 2u(\sigma_{1} \sigma_{2} + \sigma_{2} \sigma_{3} + \sigma_{1} \sigma_{3} )] $$

4 $$ \varepsilon_{2} = \frac{1}{{\text{E}}}[\sigma_{2} - {\text{u}}(\sigma_{1} + \sigma_{3} )] $$

In the unidirectional stress state, the following can be obtained:

5 $$ \varepsilon_{3} = \frac{1}{{\text{E}}}[\sigma_{3} - u(\sigma_{1} + \sigma_{2} )] $$

6 $$ {\text{W}}_{{\text{t}}} = \frac{{\sigma^{2} }}{{2{\text{E}}}} $$

where: W_t_—elastic potential of coal, MJ/m^3^; *σ*—average in situ stress, MPa; *σ*_1_, *σ*_2_, *σ*_3_—in situ stresses along three directions, MPa; *ε*_1_, ε_2_, ε_3_—strain along three directions; μ—Poisson's ratio of coal; and E—modulus of elasticity of coal, MPa.

The above equation indicates that under the same stress, the elastic potential of layered storage of a coal seam with a low elastic modulus is high; the softer the coal seam is, the lower its elastic modulus and the higher its elastic potential. Therefore, the danger of outbursts in soft coal is notable. In the original in situ stress state, the in situ stress is the premise of the occurrence of high-pressure gas^28^. The coal seam gas pressure reflects the in situ stress borne by the coal seam. In theoretical research of outburst coal seams, the coal seam gas pressure reflects the strain.

#### Gas–stress coupling elastic potential

##### Gas characteristics

Gas in a coal seam exists not only in the adsorption (solid state) and free (gas state) states but also in the liquid state. Under normal circumstances, the overall gas characteristics indicate the adsorption and free states^28^. According to statistical data of the coal seam gas content collected and measured by our research team for coal mines in Guizhou, the analysable amount of coal seam gas at normal temperatures accounts for 64–96% of the total coal seam gas content.

The gas stored in a coal seam in the form of gas and liquid and the free gas formed in the process of gas analysis exhibit the characteristics of a fluid. Both gas and fluid are compressible, and the gas in the coal seam is highly compressible in the gas state. In the process of coal and gas outbursts, considering that the temperature slightly changes, according to Boyle's law, the transformation relationship between the volume of gas and its stress can be expressed as:

7 $$ {\text{P}}_{0} {\text{V}}_{0} = {\text{P}}_{1} {\text{V}}_{1} $$

where: P_0_—Air and gas pressure, 0.1 MPa; V_0_—Volume of gas in air, m^3^; P_1_—Coal seam gas pressure, MPa; and V_1_—Coal seam gas volume, m^3^.

Then, a unit volume of gas under a stress of 1.5 MPa can expand 15 times under standard atmospheric pressure. Therefore, during outbursts, with the sudden expansion of gas, high-pressure gas can generate a gas explosion, breaking and driving coal to be quickly discharged.

##### Gas potential

The coal body can store a large amount of gas compression energy, which plays a role in crushing the coal body, transporting the outburst energy and continuously developing the outburst towards the deep part of the coal body^4,29^. At the moment of outburst initiation, gas remains in the coal body, and the heat required for gas expansion can be provided by the coal body. At this time, the internal energy of the expanded gas can be calculated according to the isothermal process. When broken coal is separated from the coal body, the internal energy of the expanded gas follows a dynamic process and finally approaches the adiabatic process. According to thermodynamics, the internal energy of the expanded gas per unit of coal can be approximately calculated with Eq. (8)^11,13,25,29^.

8 $$ {\text{W}}_{\omega } = \frac{{{\text{p}}_{1} {\text{v}}_{1} }}{{{\text{n}} - 1}}\left[ {\left( {\frac{{{\text{p}}_{0} }}{{{\text{p}}_{1} }}} \right)^{{\frac{{{\text{n}} - 1}}{{\text{n}}}}} - 1} \right] $$

where: W_ω_—resolvable gas internal energy in coal, MJ/t; v_1_—resolvable gas amount in coal, m^3^/t; P_1_—gas pressure after outburst, MPa; P_0_—coal seam gas pressure, MPa; and n—Process index.

For the isothermal process, n = 1;

For the adiabatic process, n = 1.31 (gas); and.

For the dynamic outburst process, n = 1–1.31.

According to most outburst examples, the entire process of coal and gas outburst approaches the adiabatic process^27^. For n = 1.25, under 0.1-MPa standard atmospheric conditions, Eq. (8) can be simplified as:

9 $$ {\text{W}}_{\omega } = 0.4{\text{V}}_{1} \left( {1.58P_{0}^{0.2} - 1} \right) $$

According to the calculation equation of the coal and gas elastic potential, in the process of gas outburst, the main energy source to promote outburst is the gas elastic potential.

## Results and discussion

### Relevant factors of the outburst coal quantity

**Figure 3:**
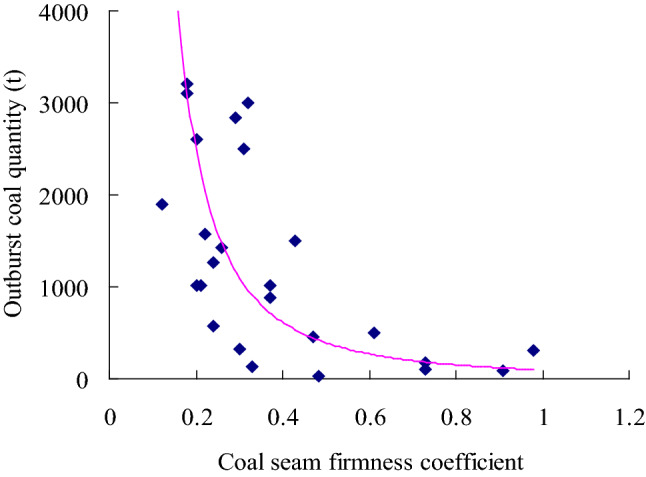
Relationship between the outburst coal quantity and coal seam firmness.

**Figure 4:**
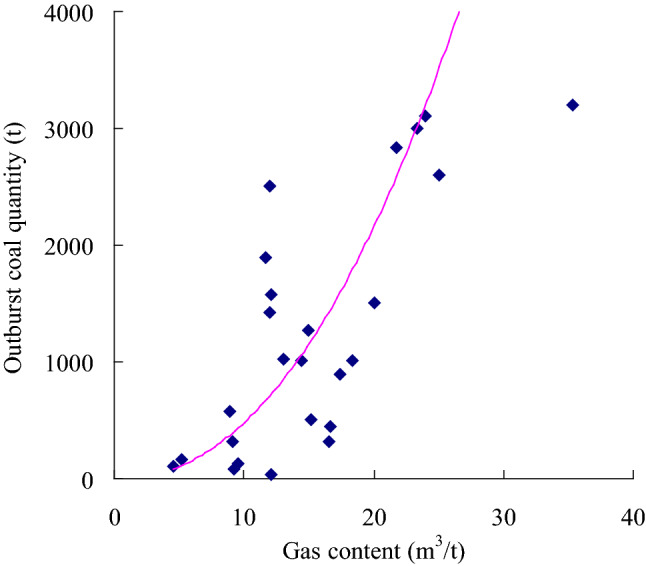
Relationship between the outburst coal quantity and coal seam gas content.

**Figure 5:**
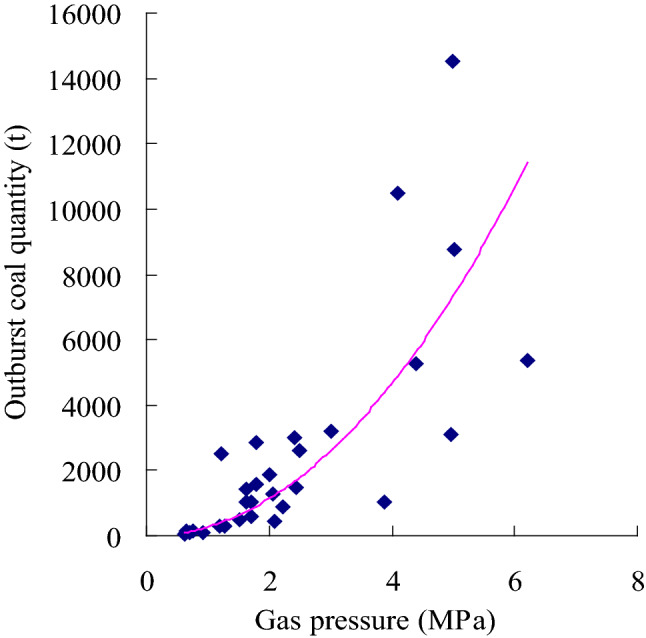
Relationship between the outburst coal quantity and coal seam gas pressure.

Based on the data summarized in Table 1, a relationship between the firmness coefficient *f* and the damage degree of structural coal was established (Fig. 3). The lower the firmness coefficient, the more developed the structural coal is, the easier the coal seam experiences outburst and the larger the outburst scale is. The firmness coefficient is related to the coal outburst amount of the coal seam. Figure 4 shows the relationship between the coal seam gas content and outburst coal quantity. According to this figure, the coal seam gas content could promote the transportation of coal seam particles in the outburst process. The higher the coal seam gas content, the more potential energy (elastic potential) can be accumulated through the coal seam gas pressure *P* and the greater the coal and gas outburst amount is. Figure 5 shows that the coal seam gas pressure is the power source of coal seam outbursts. The higher the gas pressure is, the higher the elastic potential of the coal seam and gas and the larger the outburst scale, which is consistent with findings reported in the literature^11^.

According to single-factor regression analysis of the data listed in Table 1, regression equations of the coal seam firmness coefficient, coal seam gas content, coal seam gas pressure and outburst coal volume were obtained, as provided in Eqs. (10)–(12).

The regression equation between the outburst coal quantity and coal seam firmness coefficient is:

10 $$ {\overline{\text{y}}} = 96.659f^{2.0209} \;({\text{R}}^{2} = 0.5715) $$

The regression equation between the outburst coal quantity and coal seam gas content is:

11 $$ \overline{y} = 3.1846{\text{W}}^{2.1757} \;({\text{R}}^{2} = 0.4594) $$

The regression equation between the outburst coal quantity and coal seam gas pressure is:

12 $$ {\overline{\text{y}}} = 275.28P^{2.0408} \;({\text{R}}^{2} = 0.7726) $$

According to the regression analysis results, the correlation coefficient R between the coal seam gas pressure *P* and the outburst coal quantity y is 0.88, and these factors are highly correlated. The correlation coefficient R between the coal seam firmness coefficient *f* and gas content *W* and the outburst coal quantity y are 0.75 and 0.68, respectively, and the correlation between these factors and the outburst coal quantity is significant. Theoretical analysis, empirical research and the statistical analysis data in this paper indicate that structural coal containing gas could cause coal and gas outbursts under the action of the gas pressure. On the one hand, the coal seam gas pressure plays a vital role in the initiation of coal and gas outbursts^4^ and is an important factor of the gas content and coal seam outbursts^11,28^. On the other hand, structural coal exhibits lower cohesion and strength. Structural coal can more easily form a large number of crushed and small-sized particles in the outburst process than primary coal, which is of great significance to the energy supply in the outburst process^14^. These three parameters can be used to predict coal and gas outbursts.

### Solid–gas–stress coupling equation construction

Practice, statistical data and experiments of outburst-prone coal seam mining have proven that coal and gas outbursts are the result of the comprehensive action of the in situ stress (gas pressure) P, coal seam gas content W and coal body strength *f*, while other factors are related to or derived from these three indicators^4,10,11,14,28^. Among them, the failure type of a coal seam is the external manifestation of coal body strength reduction and the result of tectonic movement. The failure degree of the coal seam reflects the strength of the coal body. Therefore, the failure type of the coal body can be reflected by the strength value of the coal body. In situ stress concentration occurs in the geological structure, and the ultimate effect of the tectonic stress in the coal seam is the gas pressure and the strength index of the coal seam. The initial velocity of gas emission is an expression of the coal seam firmness coefficient.

Therefore, through the coal seam gas pressure P, the coal seam firmness coefficient *f* and the coal seam gas content W, the coal seam outburst risk can be evaluated, outburst risk classification of outburst coal seams (i.e., classification of the outburst risk degree of outburst coal seams) can be conducted, and the outburst intensity of coal seams can be predicted.

The above discussion clarifies that structural coal containing gas, under the gas pressure of the coal seam, causes the coal body and gas to have a higher elastic potential, and the coal seam is more prone to outburst. The damage degree of the coal body, gas content and gas pressure of the coal seam exhibit obvious correlations with the outburst coal amount. The coupling action between coal (solid), gas (gas) and gas stress is the fundamental cause of coal and gas outbursts in coal seams. Therefore, according to the data in Table 1, the multiple regression equation is solved in SPSS software through multiple regression analysis^30^. After inputting the data provided in Table 1 into SPSS, through multiple linear regression analysis, the following multiple regression equation of the coupling between the outburst coal quantity and the firmness coefficient, gas content and gas pressure can be obtained:

13 $$ \overline{y} = 1539.594P + 13.388W - 1182.644f - 930.036 $$

where: ${\text{W}}_{\omega } { = }\frac{{{\text{p}}_{{1}} {\text{v}}_{{1}} }}{{\text{n - 1}}}{[(}\frac{{{\text{p}}_{0} }}{{{\text{p}}_{1} }}{)}^{{\frac{{\text{n - 1}}}{{\text{n}}}}} { - }1{]}$—outburst coal quantity, t; P—gas pressure, MPa; W—gas content, m^3^/t; and *f*—coal seam firmness.

This equation also clearly indicates that the gas pressure is the dominant factor of outbursts, and the higher the gas pressure is, the larger the amount of outburst coal. This point is consistent with findings reported in the literature^4,6,10,11^. In addition, the lower the firmness coefficient, the more likely coal and gas outburst accidents can occur^10,11,28^. The gas content in coal seams slightly impacts the outburst scale. Coal and gas outbursts are the result of the coupling effect of these three factors.

### Impact of the various factors on outbursts

According to the above solid–gas–stress coupling equation, combined with the coal seam outburst risk identification index and gas content value in the detailed rules for coal and gas outbursts^17^, the critical values of the other indicators of coal seam outbursts under different fixed index conditions were assessed (Table 2), and the possibility and cause of coal mine outbursts under these parameter conditions were predicted by using the measured gas geological parameters of two coal and gas outburst mines. The calculation data obtained with the solid–gas–stress coupling equation show that the coal outburst amount of the two coal seams was smaller than 0 t, and there was no outburst risk in the normal stratum block. A mine outburst could be caused by an increase in the gas pressure near the structure.

**Table 2:** Critical values of the outburst influencing factors under the different parameter conditions.

Index and case mine	Gas pressure P (MPa)	Gas content W (m^3^/t)	Coal seam firmness coefficient f	Outburst coal quantity Y (t)	Form comments
Variable f	0.75	8	0.28	0.00	P and W set (critical value of the appraisal index)
Variable W	0.75	27.39	0.50	0.00	P and f set (critical value of the appraisal index)
Variable P	0.92	8.0	0.50	0.00	W and f set (critical value of the appraisal index)
Under the condition of structural coal containing gas, prediction of the critical value of the gas pressure (in situ stress) during outbursts	0.95	4.00	0.50	0.00	Under the condition of setting W and f, the critical value of P for outburst occurrence
	0.80	4.00	0.30	0.00	
	0.97	2.00	0.50	0.00	
	0.82	2.00	0.30	0.00	
Sanjia Coal Mine in Zhijin County (high-gas mine)^3,1^	0.52	7.57	0.92	− 1116.13	Cases of coal mines not experiencing outbursts under the condition of measured coal seam gas geological parameters, but outbursts are observed near the structure
Anlong Guanglong Coal Mine (low-gas mine)^3,2^	0.42	4.82	0.374	− 661.19	

According to the prediction results listed in Table 2, the following analysis can be achieved:

1. Under the condition that the critical values of two outburst prediction factors must be determined, the critical values of the coal seam firmness coefficient and gas pressure are easy to obtain, but that of the coal seam gas content is difficult to determine.
2. Under the condition of structural coal containing gas, when the gas content W and firmness coefficient f of the coal seam are set, the coal seam easily reaches the critical gas pressure value for outburst occurrence, which can explain that in a coal mine with a low gas content, as long as structural coal is developed in the coal seam, even if the gas content in the coal seam is low, coal and gas outbursts can occur under the action of the gas pressure.
3. The cases of two outburst mines verified that as long as there are physical conditions (structural coal and gas) for an outburst in a given coal seam, the coal seam is an outburst coal seam, and as long as there are dynamic conditions (stress) for an outburst, the coal seam could experience an outburst.
4. Due to the uncertainty in fault development and the individual (point) characteristics of outburst identification points, coal seams not identified as outburst coal seams may experience outbursts due to the action of the structural stress. In regard to outburst coal seams, research on the development trend of geological structures must be strengthened, and geological structures must be explored to better prevent and control outburst accidents.

According to the established solid–gas–stress coupling equation, the measured coal seam firmness coefficient, coal seam gas content and gas pressure can be used to calculate the possible outburst coal quantity to determine whether an outburst risk exists at this point. Please refer to Table 3 for the outburst determination values. For a calculated outburst coal quantity of y ≥ 0t, the coal seam exhibits an outburst risk; when the outburst coal quantity y is less than 0t, the coal seam is not at risk of an outburst.

**Table 3:** Types of outburst mines and outburst prevention measures.

Outburst coal quantity y (t)	< 100	100–500	500–1000	≥ 1000
Coal seam outburst risk	Low	Medium	High	Extremely high
Type of outburst mine	Weak outburst mine	Medium outburst mine	Strong (serious) outburst mine	Extremely strong (severe) outburst mine
Regional outburst prevention measures implemented	Coal seam gas extraction via bedding drilling	Coal seam gas extraction via bedding drilling and through-layer drilling	Coal seam gas extraction via through-layer drilling and ground drilling; mining the protective layer	Mining the protective layer; coal seam gas extraction via ground drilling

With the use of this equation, we can quickly and accurately determine whether a given coal seam is an outburst coal seam during coal seam exploration, which can provide a scientific basis for project approval, preliminary design and guidance of coal uncovering operations during well construction and can offer a new method for the identification of coal seam outbursts.

### Classification of outburst mine types

According to the solid–gas–stress coupling equation and the outburst coal quantity in the statistical data provided in Table 1, the outburst risk of outburst coal seams can be divided into four categories: low outburst risk (outburst coal quantity y < 100 t), medium outburst risk (100 t ≤ outburst coal quantity y < 500 t), high outburst risk (500 t ≤ outburst coal quantity y < 1000 t) and very high outburst risk (outburst coal quantity y ≥ 1000 t) (Table 3). Then, according to the degree of the outburst risk, the types of outburst mines can be divided into weak outburst mines, medium outburst mines, strong (serious) outburst mines and very strong (very serious) outburst mines.

After categorization of the coal seam outburst risk degree, it is beneficial for mines to implement targeted outburst prevention measures while preventing improper or excessive outburst prevention measures.

### Outburst prevention measures according to the different conditions based on the solid–gas–stress coupling model

#### Improving the strength of structural coal

Structural coal is a necessary condition for coal seam outbursts. The strength of the coal body affects the difficulty of outburst. The lower the strength of the coal body (firmness coefficient *f*), the more prone the coal seam is to outbursts. Improving the strength of the coal body is an effective measure to prevent coal seam outbursts. For example, the Jiaxing coal mine in Guizhou adopts a metal framework to enhance the strength of the coal body when uncovering coal in crosscuts to prevent inducing outbursts in coal seams due to caving and collapse. The use of hydraulic punching and drainage of coal seam gas can reduce the supporting role of gas in coal seam pores, indirectly improve the coal strength and eliminate the occurrence of coal and gas outburst accidents^21^.

#### Coal seam gas extraction

Coal seam gas is another material basis for coal seam outbursts. The higher the coal seam gas content, the more energy is provided by gas for coal and gas outbursts under the same gas pressure and the higher the outburst risk is. Reducing the coal seam gas content can effectively reduce the risk and intensity of coal seam outbursts. Over the past ten years, with the implementation of regional coal seam gas extraction drilling measures, an increasing number of outburst mines have begun to extract coal seam gas on a large scale, and coal seam mining has been performed under the condition that the coal seam gas content should remain less than 8 m^3^/t^23^. The mine outburst type has gradually changed from outburst mines to extrusion mines, and the outburst intensity has gradually changed from medium to strong outbursts to small-scale outbursts. According to statistical data of the Ping'an Coal Mine Gas Control National Engineering Research Center Co., Ltd., and our research group, from 2015 to 2021, a county in Guizhou Province experienced a total of seven outbursts, with small-scale outbursts accounting for 71% of the total outbursts and medium-sized outbursts accounting for 29% of the total outbursts.

#### Reducing the coal seam gas pressure

##### Coal seam gas extraction

**Figure 6:**
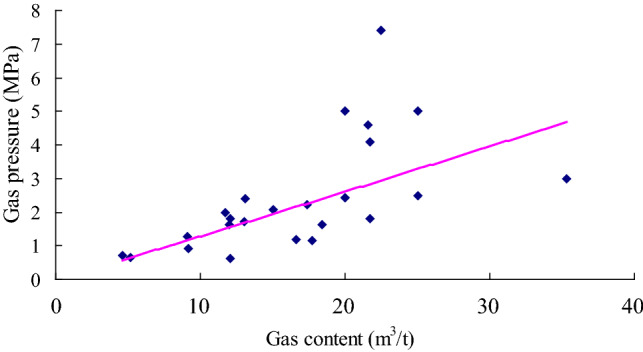
Relationship between the coal seam gas pressure and gas content.

Considering the natural occurrence state of coal seams, the relationship between the coal seam gas pressure P and gas content W is shown in Fig. 6, and the regression equation is:

14 $$ \overline{{\text{P}}} = 0.1201x^{1.0274} \;\left( {R^{2} = 0.5584} \right) $$

The correlation coefficient R was 0.75, and there occurred a significant correlation between these factors. The statistical data show that the lower the coal seam gas content is, the lower the coal seam gas pressure. Therefore, we can reduce the gas pressure of the coal seam and prevent outbursts by draining coal seam gas and reducing the gas content in the coal seam. In production practice, more than 90% of coal mines has adopted coal seam gas extraction measures, reducing the coal seam gas pressure and eliminating outbursts.

##### Mining the protective layer

**Figure 7:**
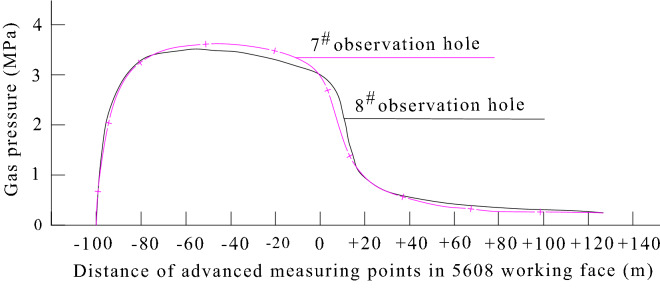
Gas pressure change in the protective B6 coal seam during mining of the 5608 protective layer working face. Both 7^#^ and 8^#^ observation holes are coal seam gas pressure observation holes installed in the B6 protected coal seam during the mining of the 5608 protective layer working face.

A large number of cases have proven that mining the protective coal seam is an effective measure to reduce the gas pressure in the protective coal seam and improve the permeability of the coal seam^33–35^. After the protective layer is mined, the gas pressure in the coal seam obviously changes. According to the research results of Yuanliang, an academician at the Chinese Academy of Engineering^35^, the gas pressure in the protected layer obviously changed before and after mining of the protective layer, and the measured minimum gas pressure was 0.2 MPa (Fig. 7). According to measured data of mining the protective layer in the Sheng'an Coal Mine, Guizhou Province, the original gas pressure of coal seam 9 in the Sheng'an Coal Mine was 1.41 MPa. After mining the protective layer of the overlying coal seam 6, the measured maximum gas pressure of coal seam 9 in the protected layer was 0.45 MPa.

## Conclusions

This paper studied a large number of outburst case data, proposed three main factors leading to outbursts, theoretically examined the main power source of coal seam outbursts, and established a solid–gas stress coupling model, which effectively overcame the shortcomings of existing outburst theories. Practice has proven that the model could provide high applicability. The main conclusions are as follows:

1. Based on an analysis of the outburst materials and energy sources, the coal seam gas content W, firmness coefficient f and gas pressure are the three leading factors of outbursts. The main power source of outburst is the elastic potential of gas. Based on the above theory, the following solid–gas–stress coupling model is established: $\overline{y}$ = 1539.594P + 13.388 W − 1182.644f − 930.036.
2. This paper explains that the main cause of low-gas mine and structural coal outbursts is the local increase in the coal seam gas pressure caused by various stresses.
3. Based on the research model established in this paper, the coal seam outburst risk is divided into four grades: low, medium, strong, and extremely high. The outburst mine types are divided into weak outburst mines, medium outburst mines, strong (serious) outburst mines, and extremely strong (very serious) outburst mines.
4. In production practice, according to the solid–gas–stress coupling model, regional outburst prevention measures such as improving the strength of structural coal, draining coal seam gas, and reducing the coal seam gas pressure can be implemented to effectively eliminate or reduce the frequency and intensity of outbursts.

## Data Availability

The datasets used and/or analysed during the current study are available from the corresponding author on reasonable request.
